# Human–Machine Telecollaboration Accelerates the Safe Deployment of Large-Scale Autonomous Robots During the COVID-19 Pandemic

**DOI:** 10.3389/frobt.2022.853828

**Published:** 2022-04-13

**Authors:** Zhongxu Hu, Yiran Zhang, Qinghua Li, Chen Lv

**Affiliations:** ^1^ School of Mechanical and Aerospace Engineering, Nanyang Technological University, Nanyang, Singapore; ^2^ Autonomous Driving Lab, Alibaba DAMO Academy, Hangzhou, China

**Keywords:** human-machine telecollaboration, hybrid intelligence, autonomous robots, COVID-19 pandemic, human-robot interaction

## Introduction

Robots are increasingly used in today’s society (Torresen, 2018; Scassellati and Vázquez, 2020). Although the “end goal” is to achieve full autonomy, currently the smartness and abilities of robots are still limited. Thus, some form of human supervision and guidance is still required to ensure robust deployment of robots in society, especially in complex and emergency situations (Nunes et al., 2018; Li et al., 2019). However, when supervising robots, human performance and ability could be affected by their varying physical and psychological states, as well as other irrelevant activities. Thus, involving people in the operation of robots introduces some uncertainties and safety issues.

Currently, the supervision of robots has become even more challenging, especially due to the COVID-19 pandemic (Feizi et al., 2021). This is because, apart from maintaining the operator’s performance, avoiding close contact between the operator and others in the working place, to keep them away from potential onsite hazards, imposes new challenges. In this context, teleoperation, which keeps humans in the control loop but at a distance, provides a solution. However, this method has limitations. Under teleoperation, continuous human supervision and control are required, resulting in a higher workload. As operators observe the environment through monitors, their fields of view are limited. They can hardly feel the motion, forces, and vibrations that the robots receive on the remote side, which further restricts their situational awareness during supervision and affects the safe deployment of robots.

**FIGURE 1:**
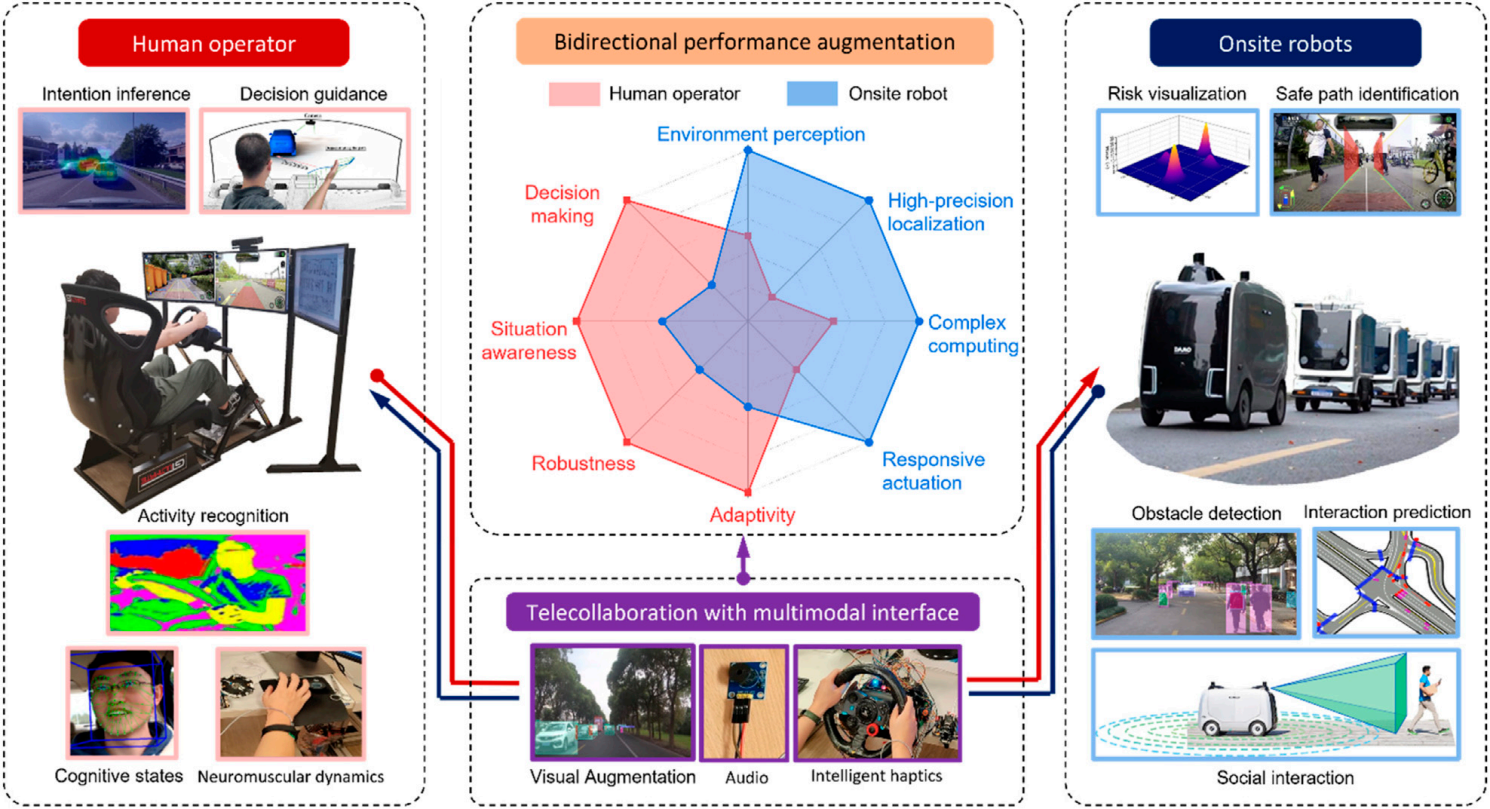
The high-level architecture of the human–robot telecollaboration system.

To overcome the above limitations and further advance safe robot operation, we propose a human–machine telecollaboration paradigm that features bidirectional performance augmentation with hybrid intelligence. In contrast to the existing teleoperation architecture, where a robot only receives and executes human commands in a single direction, the telecollaboration, as shown in Figure 1, fuses and leverages the hybrid intelligence from both human and robot sides, and keeps human operators away from onsite hazards, thereby improving the overall safety, efficiency, and robustness of the human-robot system, enabling the robust deployment of large-scale autonomous robots in society. This technology has been successfully implemented in over 200 Alibaba’s autonomous delivery robots. They are now serving more than 160 communities across 52 cities in China during the COVID-19 pandemic (Alibaba Group’s). With remote help from human operators, one robot can deliver up to 300 packages with more than 40 million obstacle detections and over 5,000 interactions per day. Recently, the fleet of delivery robots reached its milestone of one million parcel deliveries. The key features of the telecollaboration framework are as follows.

## Human–Machine Bidirectional Augmentation Framework

As both humans and machines have their strengths and weaknesses, there are no fixed roles of master and slave under this telecollaboration framework. Operators and machines complement and augment each other via multimodal interfaces for perception, decision-making, planning, and control. The performance and status of both humans and robots are monitored and assessed simultaneously in real time. The overall control authority is adaptively allocated to or fully transferred between humans and machines according to their states. Because the robots’ ability is enhanced under human remote guidance, they can function normally most of the time. Operators would need to intervene only when necessary, which reduces their workloads as full-time engagement, is not required. Ideally, one operator can supervise multiple robots simultaneously via telecollaboration. From another perspective, owing to the help received from humans, the requirements of onboard hardware and the complexity of algorithms used by the robot can be lowered. This promotes the low-cost and large-scale deployment of robots with human–robot mutual trust in society.

During human-machine collaboration, there might be conflicts happening due to deviations between human operator’s and machine’s different intentions and expectations. To address this issue and mitigate the human-machine conflicts, under the proposed telecollaboration framework, we further designed a reference-free control algorithm, in which the status, abilities, as well as intentions of the human operator and robots are monitored separately (Huang et al., 2020). And based on their individually assessed performances, the entire control authority is then allocated to each party accordingly, ensuring the safety, stability and smoothness of the overall human-machine system.

## Augmented Machine Intelligence Under Human Guidance

Robots, not pure mechanical actuators, have a certain degree of smartness and can autonomously operate under normal conditions. However, because of their limited abilities, robots can face considerable challenges in complex situations, which they may not be able to handle by themselves (Rossi et al., 2020). In such situations, human intervention is needed to provide guidance and compensation via telecollaboration at either the strategic or tactical level. Through multimodal interfaces, including image processing, gesture recognition, natural language processing, and affective computing, the operator’s intentions can be inferred and passed to the machines to enhance their reasoning (Yang et al., 2018). Recently, we proposed a novel human–machine cooperative rapidly exploring random tree algorithm, which introduces human preferences and corrective actions, enhancing the safety, smoothness, and human likeness of robot planning and tracking control (Huang et al., 2021). In addition, to further improve machine intelligence, we are developing a human-in-the-loop deep reinforcement learning (DRL) method by fusing human skills via real-time guidance into the DRL agent during the learning process, which effectively improves the learning curve (Wu et al., 2021).

## Augmented Human Performance Leveraging Machine Intelligence

Compared to robots, humans exhibit stronger robustness and adaptability in scene understanding and decision-making; however, their abilities are limited in areas such as high-precision perception, localization, measurement, and complex computing (Fani et al., 2018; Su et al., 2020). In particular, under remote collaboration, the human field of view and subjective feelings are restricted. Fortunately, these deficiencies can be compensated and augmented by machines (Drnach and Ting, 2019). Multi-modal and multi-dimensional onsite information, including simultaneous localization and mapping, obstacle detection, and potential risk assessment, can be processed by onsite robots using onboard sensors, sent back via telecommunications, and visualized on monitors to improve the operator’s situational awareness. In addition, being deployed in society, robots will need to interact with humans. Therefore, interaction awareness and forecasting are important for safe robot operations. Recently, we proposed a novel deep learning model that uses only one second of historical data to accurately predict the robot motion and its interactions with surrounding agents up to 8 s ahead, wining the first Place of Interaction Prediction Track at 2021 Waymo Open Dataset Challenges (Mo et al., 2022). The visualized interaction prediction results effectively help operators understand the situation and make safe decisions. In addition, multimodal interfaces, including computer vision, augmented reality, virtual reality, electromyography, and haptics, have been explored to help identify the operator’s cognitive and physical states and allow automation to provide personalized assistance, thereby improving the operator’s task ability during telecollaboration (Lv et al., 2021).

## Conclusion

The large-scale real-world robot deployment results demonstrated the feasibility and effectiveness of the proposed telecollaboration. The high-level framework, submodules, and algorithms that were developed have great potential to be expanded to a wide range of robotics and human–machine interaction applications. Nevertheless, social robot deployment is a system-engineering task that requires collaboration between different areas, such as AI, robotics, control, telecommunications, human factors, psychology, privacy, security, ethics, and law. In the future, more efforts from multi-disciplinary aspects, including human-like autonomy, cognitive robotics, multi-modal human-machine interface, low-cost perception, high-definition map, 5G communication, cloud and edge computing, safe AI etc, need to be made to develop a harmonic ecosystem for human–robot coexisting societies.
